# Evaluating the necessity of booster sessions in relapse prevention for depression: a longitudinal study

**DOI:** 10.3389/fpsyg.2025.1568141

**Published:** 2025-06-24

**Authors:** Jón Ingi Hlynsson, Tómas Kristjánsson, Gerhard Andersson, Per Carlbring, the ACTUA research group

**Affiliations:** ^1^Department of Psychology, University of Iceland, Reykjavík, Iceland; ^2^Department of Psychology, Stockholm University, Stockholm, Sweden; ^3^Department of Behavioural Sciences and Learning, Linköping University, Linköping, Sweden; ^4^Department of Biomedical and Clinical Sciences, Linköping University, Linköping, Sweden; ^5^Department of Clinical Neuroscience, Karolinska Institute, Stockholm, Sweden; ^6^School of Psychology, Korea University, Seoul, Republic of Korea

**Keywords:** major depression, behavioral activation, physical activity, relapse prevention, treatment booster, survival analysis

## Abstract

**Introduction:**

Major depression is a highly prevalent and heterogenous mental disorder. Although therapeutic advances for major depressive disorder over the past quarter-century have been incremental rather than transformative, booster sessions have been proposed as a means of solidifying acute treatment gains and lowering relapse risk. However, evidence for the effectiveness of these treatment booster sessions remains inconclusive. This study therefore evaluated the long-term effectiveness of relapse prevention treatment booster sessions for major depression.

**Method:**

In a two-arm, parallel-group, maintenance-phase randomized controlled trial (RCT) with repeated longitudinal measures, the sample consisted of participants in Sweden who had received acute treatment for depression (internet-based behavioral activation or physical activity) and were then randomly assigned to either an 8-week relapse prevention program (*n* = 119) or control group (*n* = 143). Participants were followed-up for 24-months with both monthly self-report questionnaires (Patient Health Questionnaire 9-item & Generalized Anxiety Disorder 7-item) and quarterly diagnostic interviews (Mini-International Neuropsychiatric Interview; MINI).

**Results:**

Both the relapse prevention group and control group exhibited similar depression-free trends over the course of the study period, with over 95% of participants in each group maintaining remission at the 24-month follow-up. Furthermore, all pre-hypothesized predictors of relapse were non-significant in differentiating the two groups at 24-month follow-up.

**Discussion:**

These findings raise the question of whether treatment booster sessions are uniformly advisable for all mild–moderate cases of depression. For instance, preferentially recommending treatment boosters for psychotherapy-naïve individuals with depression may yield greater effects compared to individuals with difficult-to-treat depression. Our findings indicate that the efficacy of behavioral activation and physical activity may be even greater than previously reported, a testament to the lasting effects of internet-based psychotherapy.

**Clinical trail registration:**

ClinicalTrials.gov, identifier NCT01619930.

## 1 Introduction

Major depressive disorder (MDD) is a highly prevalent mental disorder. Global prevalence estimates indicate that 279.6 million people currently suffer from a depressive disorder (GBD 2019 Mental Disorders Collaborators, 2022), with 12-month prevalence rates for MDD estimated at 7% (American Psychiatric Association, 2022). In Sweden, around 10.8% of the population is estimated to suffer from clinically significant depression at any given time point (Johansson et al., 2013). Furthermore, the economic toll depressive disorders impose on modern societies has become increasingly high; annual economic burden increased by 37.9% between 2010 and 2018 (Greenberg et al., 2021). However, our understanding of depression remains limited by its heterogeneity (Goldberg, 2011) and underdeveloped explanatory theory-driven models (Fried et al., 2022). For instance, 10,377 different symptom presentations can be derived from the nine symptoms of major depressive disorder (Fried et al., 2020). Additionally, depression has too often been conflated with simple sadness or bereavement (Wakefield et al., 2017). Recommended treatments for mild–moderate major depression include cognitive-behavioral therapy, behavioral activation, and physical activity such as group exercise (National Institute for Health and Care Excellence [NICE], 2022).

Given the heterogeneous nature of depression and underdeveloped theory-driven models to underpin treatment, it is unsurprising that treatment outcomes for depression have remained stable for the last 25 years (Cuijpers et al., 2024). However, an exploration of the treatment modalities that have shown promise and are recommended by clinical guidelines (i.e., behavioral activation and physical activity; National Institute for Health and Care Excellence [NICE], 2022) may provide insights into where to begin conceptualizing further developments of the treatment of depression. Additionally, some evidence suggests that treatment booster sessions following acute treatment for depression solidify treatment gains, with findings that mirror conventional therapy (Bruin et al., 2022; Clarke et al., 2015). However, evidence for their effectiveness remains unclear, with studies showing mixed results in preventing relapse (e.g., Hadjistavropoulos et al., 2022; Klein et al., 2018; McCartney et al., 2021).

### 1.1 Relapse prevention treatment booster sessions for depression

Although the addition of booster sessions has shown promise to solidify treatment gains in treatment of depressive disorders (Bruin et al., 2022), the evidence for their efficacy is still inconclusive (McCartney et al., 2021). This is especially true within the internet-based therapy literature. For instance, recent studies on internet-based therapy for depression have not found treatment booster sessions to improve client outcomes on measures of depressive symptom severity (Hadjistavropoulos et al., 2022; Peynenburg et al., 2022). Furthermore, interest in receiving treatment booster sessions has been associated with lower pre-treatment symptom severity (Hadjistavropoulos et al., 2022), in turn indicating that those whom might benefit most from treatment boosters are those whom do not show interest in them (Patterson et al., 2022). Nonetheless, meta-analytic findings do suggest that the risk of relapse is generally reduced by over 20% for clients who receive treatment booster sessions up to 24-months following treatment, as compared to clients receiving treatment as usual (Bruin et al., 2022; Clarke et al., 2015).

Analogous findings have also been found within the internet-based therapy literature. For instance, among iCBT-naïve participants with remitted depression recruited in a risk of relapse following previous MDD treatment, the risk of relapse is significantly reduced following treatment booster sessions (Holländare et al., 2011). Holländare et al. (2011) found this lower risk to be mediated by the improvement of depressive symptoms. Conversely, they also found residual post-treatment symptoms of depression to predict relapse. Preventive internet-based therapy for individuals with remitted but recurrent depression has not been shown to be effective and cost-effective compared to treatment as usual (Klein et al., 2018). Although preventive therapy is not equivalent to treatment booster sessions, these findings cast doubt on the effectiveness of relapse prevention using internet-based interventions. Furthermore, relatively few studies have evaluated internet-based treatment booster sessions for depression.

Taken together, although treatment booster sessions have shown promise in the internet-based therapy literature (Holländare et al., 2011) with findings that mirror conventional therapy (Bruin et al., 2022; Clarke et al., 2015), the lack of studies on internet-based treatment boosters for depression, as well as and the inconsistent results evident in the relapse prevention literature (Hadjistavropoulos et al., 2022; Holländare et al., 2011; Klein et al., 2018; Peynenburg et al., 2022) make it difficult to ascertain their effectiveness.

### 1.2 Behavioral activation

Behavioral activation is a brief and structured form of psychotherapy that aims to increase engagement in adaptive activities (i.e., activities associated with experiencing pleasure or mastery; Dimidjian et al., 2011; Martell et al., 2021). Considering the heterogeneity of depression (Fried et al., 2020; Goldberg, 2011), it is crucial to individualize behavioral activation for each client (Dimidjian et al., 2021; Martell et al., 2021). Thus, behavioral activation can be considered as a bespoke treatment approach that takes into account the unique case formulation of each client and tailors adaptive activation accordingly. Primary aims of behavioral activation are threefold: (a) increasing engagement in activities associated with pleasure and mastery, (b) decreasing engagement in activities that maintain or increase risk of depression, and (c) problem-solving hurdles that limit access to rewarding stimuli or increase aversive control (i.e., using aversive stimuli or consequences like punishment or negative reinforcement to control behavior; Dimidjian et al., 2011).

Behavioral activation can also be delivered via the internet without compromising therapeutic efficacy (Hedman-Lagerlöf et al., 2023). Internet-based behavioral activation has been supported with meta-analytic findings showing a moderate overall effect size of treatment efficacy (Alber et al., 2023; Huguet et al., 2018). Although internet-based behavioral activation consistently performs as well or better than other treatment outcomes and control groups (Fu et al., 2021; Ly et al., 2014; Nyström et al., 2015), most studies lack sufficient follow-up measurements to adequately ascertain the long-term efficacy of internet-based behavioral activation (Huguet et al., 2018). However, emerging evidence suggests that internet-based cognitive behavioral therapy can have enduring effects, with improvements maintained for up to five years in some cases (Andersson et al., 2018). Moreover, a preponderance of the evidence to date relies exclusively on self-report measures of depression, as compared to clinical interviews (Alber et al., 2023; Huguet et al., 2018). As such, there is a need for high-quality longitudinal studies that evaluate the long-term effectiveness of internet-based behavioral activation to increase confidence in the above-mentioned findings.

### 1.3 Physical activity

Group exercise and physical activity are among the recommended treatments of choice for mild–moderate major depression (National Institute for Health and Care Excellence [NICE], 2022). Meta-analytic findings and systematic reports suggest that physical activity can have a significant antidepressant effect (Heissel et al., 2023; Hird et al., 2024; Kvam et al., 2016; Morres et al., 2019; Nyström et al., 2015; Rebar et al., 2015). For instance, physical exercise reduces the levels of pro-inflammatory factors and increases the levels of anti-inflammatory factors, which has been suggested to be beneficial for preventing the occurrence of major depression (Cui et al., 2024; Hird et al., 2024). The combination of high-intensity physical activity such as aerobic exercise and low-intensity physical activity such as yoga and stretching have shown to have a significant antidepressant effect among individuals with depression (Imboden et al., 2020). Moreover, a dose-dependent relationship has been found between daily step count and depression improvement (Dang et al., 2023); although with the caveat of a small sample size.

Notably, physical activity appears to have lasting antidepressant effects that are maintained up to 12-months following treatment (Helgadóttir et al., 2017; Wolf et al., 2024). Moreover, a recent study comparing physical exercise and behavioral activation found comparable effects on depressive symptom severity among individuals with mild to moderate levels of depression (Nyström et al., 2017), in turn further solidifying physical exercise as a viable treatment for major depression. Taken together, physical exercise appears to have lasting antidepressant effects which are comparable to previously validated behavioral treatments.

### 1.4 Moderators of treatment outcomes in previous studies

Several variables have been found to moderate treatment outcome predictors in previous studies. For instance, higher pre-treatment levels of depressive symptom severity predict treatment response to internet-based therapy for depression (Haller et al., 2023; Webb et al., 2017). Furthermore, (increasing) age (Rozental et al., 2017), higher educational attainment (Ebert et al., 2016), marital status (specifically being separated, widowed, or divorced; Button et al., 2012), and female gender (Donker et al., 2013) predict greater depressive symptom improvement in internet-based therapy studies (Rozental et al., 2017; Webb et al., 2017; for a review of moderators in internet-based interventions, see Haller et al., 2023). Additionally, greater treatment response has been associated with a simultaneous reduction in anxiety symptom severity and an improvement in quality of life ratings (Alber et al., 2023; Ly et al., 2014). Furthermore, comorbid anxiety has also been associated with worse treatment outcomes (Haller et al., 2023). Finally, previous findings indicate that “difficult-to-treat depression” (DTD) modulates treatment response and risk of relapse (Rush et al., 2022). A recent consensus statement on DTD proposed the following definition: “*depression that continues to cause significant burden despite usual treatment efforts*” (McAllister-Williams et al., 2020, p. 266). The key characteristic of DTD is the failure of prior treatment attempts (including both psychotherapy and pharmacotherapy) to alleviate the depressive symptomatology (McAllister-Williams et al., 2020; Rush et al., 2022). This, in turn, suggests that treatment outcome studies should adjust for indicators of DTD in analyses of treatment outcomes in depression treatment studies.

Taken together, existing findings suggest that analyses depression relapse must take into consideration pre-treatment levels of depression, anxiety levels, quality of life, age, education level, marital status, and gender, as well as whether participants have previously undergone psychotherapy or pharmacotherapy.

### 1.5 Aims of the current study

This study aims to evaluate the long-term efficacy of internet-based behavioral activation and physical activity, assess factors that are predictive of relapse prevention, and evaluate the effectiveness of the relapse prevention program. As a follow-up to a previous treatment study (Nyström et al., 2017), participants were randomly assigned to either a relapse prevention booster group or a control group that does not receive a booster following acute treatment.

We hypothesized that allocation to a relapse prevention treatment booster group would be associated with a reduced risk of relapse. Additionally, we hypothesized that lower pre-booster levels of depression and anxiety, higher quality of life, older age, higher education level, being in a relationship, female gender, and a history of prior psychotherapy or pharmacotherapy would be negatively associated with relapse at the 12-month and 24-month post-assessments.

## 2 Method

### 2.1 Participants and recruitment

Participants were recruited in Sweden through social media and advertisements in newspapers. Participants were first screened for eligibility in the acute treatment study (described in detail elsewhere, see Nyström et al., 2017). Eligible participants were randomly assigned to treatment groups consisting of a variation of either behavioral activation or physical activity. Following this acute treatment, participants were then randomly assigned to either a treatment booster group or a control group. The present study’s foci is directed on the follow-up to acute treatment; detailed information about the original study, such as pre-acute treatment sample characteristics information, is detailed elsewhere (Nyström et al., 2017). This study included 262 participants in total, 119 participants in the treatment booster group and 143 in the control group (see Table 1).

**Table 1:** Demographic characteristics and treatment outcome comparisons for booster and control groups.

	Booster (*n* = 119)	Control (*n* = 143)	*p*-value
Age: M (SD) [min-max]	41.86 (12.70) [20.00–76.00]	40.94 (13.06) [20.00–80.00]	0.565
Sex: *N* (%)			0.156
Female	96 (81%)	103 (73%)	
Male	23 (19%)	39 (27%)	
Civil status: *N* (%)			0.732
Single	23 (33%)	28 (30%)	
In a relationship	40 (57%)	54 (58%)	
Divorced	7 (10%)	11 (12%)	
Education level: *N* (%)			0.591
Elementary	5 (7%)	10 (10%)	
High school	25 (35%)	29 (30%)	
Graduate school	40 (56%)	53 (55%)	
Postgraduate	2 (3%)	5 (5%)	
Prior psychotherapy: *N* (%)			
Yes	72 (61%)	79 (56%)	0.517
No	47 (39%)	63 (44%)	
Prior Pharmacotherapy: *N* (%)			0.398
Yes	58 (49%)	61 (43%)	
No	61 (51%)	81 (57%)	
Measures: M [95% CI]			
Pre-treatment^a^			
PHQ-9	14.72 [14.03; 15.41]	14.27 [13.59; 14.96]	0.363
GAD-7	9.85 [9.07; 10.63]	10.59 [9.82; 11.36]	0.181
QOLI	−0.68 [−0.95; −0.41]	−0.76 [−1.03; −0.50]	0.656
Post-treatment^b^			
PHQ-9	7.35 [6.22; 8.48]	7.25 [6.09; 8.41]	0.903
GAD-7	5.63 [4.68; 6.57]	5.54 [4.56; 6.53]	0.904
QOLI	0.57 [0.20; 0.93]	0.65 [0.27; 1.02]	0.759
12-month follow-up			
PHQ-9	6.25 [4.97; 7.52]	5.46 [4.28; 6.64]	0.367
GAD-7	4.80 [3.77; 5.83]	4.15 [3.18; 5.12]	0.358
QOLI	1.09 [0.60; 1.59]	0.99 [0.53; 1.45]	0.760
24-month follow-up			
PHQ-9	6.29 [4.97; 7.61]	5.75 [4.34; 7.17]	0.584
GAD-7	4.54 [3.56; 5.52]	3.93 [2.91; 4.96]	0.394
QOLI	0.95 [0.57; 1.34]	1.18 [0.77; 1.58]	0.426

Numerical variables are reported as mean (SD) [range: minimum-maximum]. Categorical variables are reported as count (percentage). Data were missing for age from 1 participant in the control group, for gender from 1 participant in the control group, for remission from 36 participants in the booster group and 51 in the control group, for civil status from 49 participants in the booster group and 50 in the control group, for education level from 47 participants in the booster group and 46 in the control group, for prior psychotherapy from 1 participant in the control group, and for prior pharmacotherapy from 1 participant in the control group. For pre-treatment measures, data were missing for 1 participant in the control group for PHQ-9, GAD-7, and QOLI. For post-treatment measures, data were missing from 36 participants in the booster group and 51 in the control group for PHQ-9 and GAD-7, and from 40 in the booster group and 53 in the control group for QOLI. For 12-month follow-up, data were missing from 58 participants in the booster group and 69 in the control group for PHQ-9 and GAD-7, and from 61 in the booster group and 70 in the control group for QOLI. For 24-month follow-up, data were missing from 56 participants in the booster group and 70 in the control group for PHQ-9 and GAD-7, and from 56 in the booster group and 71 in the control group for QOLI. PHQ-9, Patient Health Questionnaire 9-item; GAD-7, Generalized Anxiety Disorder scale 7-item; QOLI, Quality of Life Inventory.

a Baseline scores prior to acute treatment.

b Baseline scores prior to relapse prevention program allocation.

### 2.2 Study design

This maintenance-phase randomized controlled trial used a two-arm, parallel-group design with repeated longitudinal assessments. Following acute treatment (described in detail elsewhere, see Nyström et al., 2017), participants were randomized 1:1 to either a relapse-prevention booster or usual-care control. An *a priori* power analysis (*α* = 0.05, 80% power) indicated that 500 participants (250 per arm) would detect a small-to-moderate effect on the primary outcome (see Carlbring et al., 2013). Thus, although the acute treatment study included four treatment groups (and a control group), the present follow-up study can be thought of as comprising of two groups consisting of those whom either received or did not receive a relapse prevention treatment booster (see Supplementary Figure S1 for a flow diagram of the recruitment process and group allocation). The benefit of conceptualizing the study population as a two-group sample is nested in the increased statistical power achieved with larger group sizes. Moreover, any variation in the four preceding treatment conditions is balanced by the randomization into booster/non-booster conditions.

Follow-up measurements were collected 24-months following treatment using a combination of survey questionnaires and follow-up diagnostic interviews. Participants were prompted to answer the PHQ-9 (Kroenke et al., 2001) and GAD-7 (Spitzer et al., 2006) each month post-treatment. Diagnostic interviews (i.e., the depression chapter in the Mini-International Neuropsychiatric Interview (MINI); Sheehan et al., 1998) were also conducted on a group level every month. However, on an individual level, these diagnostic interviews were conducted every third month during the 24-month follow-up period. Stated differently, 33% of each treatment group from the acute treatment allocation was interviewed via telephone each follow-up month. In turn, eight follow-up interviews were conducted in total for each participant and the diagnostic interviews have a built-in data-loss of 66% each month. This built-in data-loss of 66% each month was, however, data missing completely at random (MCAR) as the participants were randomized to their respective months, making the procedure unbiased.

### 2.3 Treatment interventions

A brief description of the relapse prevention program follows. Following acute treatment, 50% of each treatment group was allocated to a relapse prevention program at random. The relapse prevention program consisted of eight treatment booster sessions that were delivered over eight consecutive weeks. These modules included techniques such as cognitive restructuring to challenge negative thought patterns, relaxation exercises to manage stress, and mindfulness practices to promote present-moment awareness. Participants were also encouraged to engage in regular physical activities, emphasizing consistency rather than intensity. The program provided psychoeducation on the importance of maintaining healthy routines, including regular sleep and nutrition. Additionally, goal-setting exercises were implemented to help participants identify and pursue meaningful activities, fostering a sense of purpose and motivation. These elements aimed to reinforce skills learned during the initial treatment phase, thereby reducing the risk of relapse.

### 2.4 Measures

#### 2.4.1 The MINI-international neuropsychiatric interview (MINI)

The MINI is a brief and structured diagnostic interview that evaluates psychiatric disorders according to the DSM-IV and ICD-10 diagnostic criteria (Sheehan et al., 1998). The MINI has been used as the gold-standard indicator of a depression diagnosis in previous studies (e.g., Nejati et al., 2020), although a recent synthesis of three large meta-analyses indicated that the MINI may be more likely to diagnose depression than other structured diagnostic interviews (Wu et al., 2020). This aligns with the intended function of the MINI as it was designed to be over-inclusive screening interview for classifying psychiatric disorders (Sheehan et al., 1998). Previous studies have found the MINI to be a reliable and valid screening tool (Lecrubier et al., 1997; Sheehan et al., 1997). In this study, interviewers administering the MINI interviews were blinded to the participants’ treatment allocation.

#### 2.4.2 Patient health questionnaire 9-item scale (PHQ-9)

The PHQ-9 is a self-report questionnaire consisting of nine items that quantify depressive symptom severity (Kroenke et al., 2001). Designed to capture all nine symptoms of major depression, the PHQ-9 routinely demonstrates good accuracy and discrimination ability in both clinical settings and the general population (Hlynsson and Carlbring, 2024; Martin-Key et al., 2022). A total score of 10 or higher is considered a diagnostic indicator for depression (Kroenke et al., 2001, 2010). In this sample, the internal consistency reliability for the PHQ-9 was good at post-assessment, Cronbach’s *α* = 0.88 [95% CI: 0.86, 0.90].

#### 2.4.3 Generalized anxiety disorder 7-item scale (GAD-7)

The GAD-7 is a self-report questionnaire consisting of seven items that quantify anxiety symptom severity (Spitzer et al., 2006). Although initially designed to index symptoms of generalized anxiety disorders, the GAD-7 is sensitive to various anxiety disorders (Kroenke et al., 2010) and has demonstrated good accuracy and discrimination ability in both clinical settings and the general population (Hlynsson and Carlbring, 2024; Martin-Key et al., 2022). A total score of 8 or higher is considered a diagnostic indicator for an anxiety disorder (Luo et al., 2019). In this sample, the internal consistency reliability for the GAD-7 was good at post-assessment, Cronbach’s α = 0.89 [95% CI: 0.87, 0.91].

#### 2.4.4 Quality of life inventory (QOLI)

The Quality of Life Inventory (QOLI) is a 32-item self-report questionnaire that indexes self-perceived quality of life across 16 domains (Frisch et al., 1992). The QOLI is unique in that it evaluates not only satisfaction with a certain aspect of life but also how important that aspect is for well-being (although other instruments have also adopted this weighted satisfaction approach, see Lindner et al., 2016). The 32 items are rated on a 3-point rating scale for importance, and 6-point rating scale for satisfaction 16 domains (Frisch et al., 1992). Thus, the QOLI produces an overall quality of life score and a weighted satisfaction profile for the 16 areas of life that make up the overall score. Previous studies have shown the QOLI to be to be a reliable and valid indicator of quality of life that exhibits excellent test–retest reliability (Thorndike et al., 2009) and is adequately responsive to change (Lindner et al., 2013; Thomas et al., 2012). In this sample, the internal consistency reliability for the QOLI was good at post-assessment, Cronbach’s α = 0.82 [95% CI: 0.79, 0.85].

### 2.5 Data analysis

Data analysis was conducted using R (R Core Team, 2021). To assess the effectiveness of the relapse prevention program, the Kaplan–Meier estimator (Goel et al., 2010) and *survminer* package (Kassambara et al., 2021) was be used to estimate survival rates (i.e., time until relapse as measured by the MINI follow-up interviews). This method has been used in previous studies with analogous data (Mueller et al., 2004; Voorhees et al., 2020). Two dates were missing in the MINI follow-up interview data which were imputed using linear interpolation, as data for dates were on a linear time scale with known dates for data preceding and following the interpolated date (Friedman, 1962). As this study aimed to evaluate the 24-month survival rates for time without relapse, only data that were collected in that 24-month time period was used in the analyses; 8 interviews were conducted after the 24-month time span and were subsequently removed from the dataset.

Given the design of the present study, using the MINI interview to evaluate predictors of treatment outcomes is unfeasible. Instead, the PHQ-9 will be used as a proxy indicator for depression status at 12-month and 24-month follow-up measurements. To that aim, logistic regression analyses on background variables will be performed to evaluate potential predictors of relapse prevention. Hosmer and Lemeshow's (1980) goodness-of-fit test was used to evaluate model fit of the logistic regression models, with *p* < 0.05 indicating an optimal fit. It should be noted that due to the few number of participants that reported being divorced, marital status was entered as a numeric variable in the regression models. Education was also treated as a numeric variable with the same rationale in the logistic regression models. As this study aims to evaluate the effectiveness of the relapse prevention program (not acute treatment effects), pre-treatment levels of depression, anxiety, and quality of life refer to pre-booster treatment (corresponding to post-acute treatment); acute treatment results are detailed elsewhere (Nyström et al., 2017).

## 3 Results

In total, the sample consisted of 262 participants; 119 participants were allocated to the relapse prevention treatment booster group and 143 to the control group. No significant differences were found between the treatment booster group and the control group on relevant background variables (see Table 1).

### 3.1 Survival analysis

To estimate whether survival rates (i.e., time until relapse) differed as a function of treatment group allocation, the depression status for each follow-up interview by treatment group was inspected. In total, 34 occurrences of depression were detected in the treatment booster group and 42 occurrences of depression were detected in the control group. See Table 2 for a delineation of depression status for each MINI follow-up interview by treatment group allocation.

**Table 2:** Depression status by follow-up interview number and intervention group.

MINI follow-up interview number	Depression status	Booster (*n* = 119)	Control (*n* = 143)
Follow-up interview one	Not Depressed	79 (91.9%)	86 (95.6%)
	Depressed	7 (8.1%)	4 (4.4%)
Follow-up interview two	Not Depressed	71 (88.8%)	85 (95.5%)
	Depressed	9 (11.3%)	4 (4.5%)
Follow-up interview three	Not Depressed	72 (96.0%)	75 (89.3%)
	Depressed	3 (4.0%)	9 (10.7%)
Follow-up interview four	Not Depressed	74 (98.7%)	82 (93.2%)
	Depressed	1 (1.3%)	6 (6.8%)
Follow-up interview five	Not Depressed	72 (93.5%)	76 (93.8%)
	Depressed	5 (6.5%)	5 (6.2%)
Follow-up interview six	Not Depressed	65 (94.2%)	72 (88.9%)
	Depressed	4 (5.8%)	9 (11.1%)
Follow-up interview seven	Not Depressed	65 (97.0%)	76 (97.4%)
	Depressed	2 (3.0%)	2 (2.6%)
Follow-up interview eight	Not Depressed	62 (95.4%)	75 (96.2%)
	Depressed	3 (4.6%)	3 (3.8%)

Follow-up interview data represents aggregate group data for interviews conducted at 3-month intervals over the 24-month study duration; interview one was conducted in months 1–3, interview two in months 4–6, interview three in months 7–9, interview four in months 10–12, interview five in months 13–15, interview six in months 16–18, interview seven in months 19–21, and interview eight in months 22–24 following post-acute treatment. True missing data, not by design, increased over the course of the study and were as follows: Interview one (32.9%), two (35.5%), three (39.3%), four (37.8%), five (39.7%), six (42.7%), seven (44.7%), eight (45.4%).

**Figure 1:**
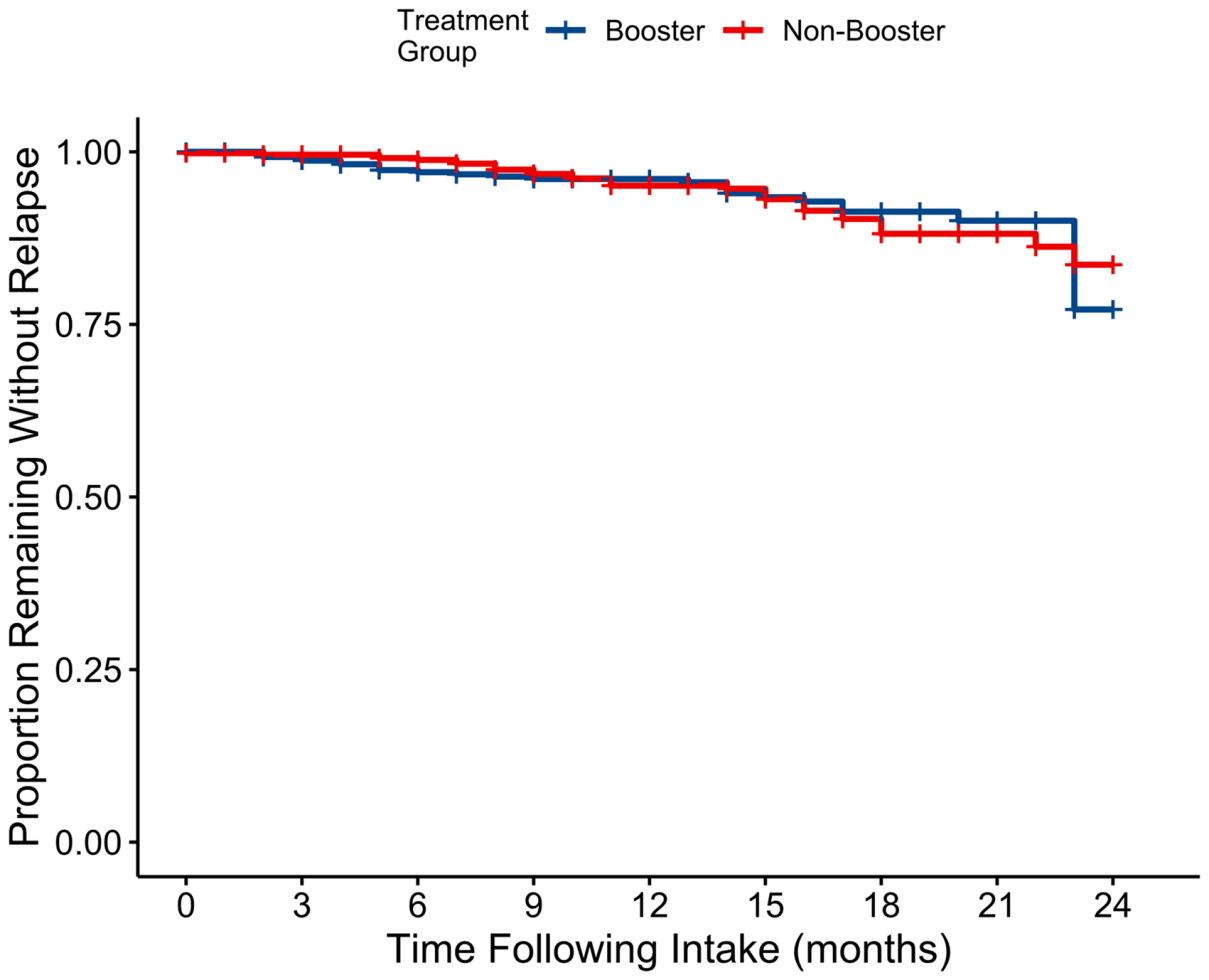
Survival curves of depression status over the follow-up period by treatment group as assessed by the MINI follow-up interviews.

Next, a survival analysis that stratified participants by treatment group allocation was performed. Figure 1 displays the survival curves for depression status over eight MINI follow-up interviews. The comparison between the relapse prevention treatment booster group (blue) and the control group (red) showed no statistically significant difference in relapse rates (*p* = 0.65). Both groups exhibited similar trends in maintaining a depression-free status over the course of follow-up measurements. As such, our hypothesis of higher relapse rates in the control group was not supported. A similar trendline was detected when survival curves were plotted using PHQ-9 monthly follow-up data (see Supplementary Figure S2 for the survival analysis and Supplementary Table S1 for arithmetic mean scores on the PHQ-9 by month). However, the survival analysis using the PHQ-9 monthly follow-up data suggested that the relapse prevention group treatment booster group and control group were significantly different. A visual inspection of the plot and monthly follow-up mean values, juxtaposed with the MINI interview data presented in Figure 1, however, does not support a clinically significant difference between these two groups.

### 3.2 Predictors of relapse prevention

To evaluate whether the hypothesized background variables pre-acute treatment levels of depression, anxiety, quality of life, age (higher), education level (higher), marital status, gender, prior psychotherapy, and pharmacotherapy were significantly predicted relapse prevention, logistic regression models were constructed for the 12-month and 24-month relapse propensity.

Several predictors were notably associated with depression status at a 12-month follow-up (see Table 3). However, only post-treatment quality of life scores emerged as a statistically significant predictor of depression status; a one-unit increase in post-treatment Quality of Life Inventory (QOLI) scores decreased the odds of being depressed at a 12-month follow-up by about 66% (*p* = 0.016).

**Table 3:** Results of the logistic regression analysis depression status at 12-month follow-up (Not-Depressed = 0, Depressed = 1).

Predictor	β	OR	*p*-value
(Intercept)	−6.16 (3.69)	0.00 [0.00; 1.48]	0.095
Treatment Group: Control	−1.16 (1.01)	0.31 [0.04; 2.17]	0.249
PHQ-9: Post-treatment	−0.09 (0.16)	0.91 [0.64; 1.19]	0.566
GAD-7: Post-treatment	0.27 (0.19)	1.31 [0.95; 2.01]	0.143
QOLI: Post-treatment	−1.08 (0.45)	0.34 [0.11; 0.73]	0.016*
Age	0.09 (0.06)	1.10 [0.99; 1.26]	0.098
Gender: male	−3.27 (1.80)	0.04 [0.00; 0.83]	0.070
Marital status (descending)	0.04 (1.00)	1.04 [0.11; 7.22]	0.971
Education level (ascending)	−0.06 (0.69)	0.94 [0.24; 3.96]	0.933
Prior pharmacotherapy: Yes	−0.09 (1.22)	0.92 [0.07; 10.66]	0.942
Prior psychotherapy: Yes	0.52 (1.19)	1.68 [0.18; 23.51]	0.662

Standard errors are displayed in parentheses; 95% confidence intervals are displayed in brackets. The model fit was good, χ2(10) = 20.8, *p* = 0.022. PHQ-9, Patient Health Questionnaire 9-item; GAD-7, Generalized Anxiety Disorder scale 7-item; QOLI, Quality of Life Inventory.

Whether or not confidence intervals include zero should not prevent meaningful interpretation of effect sizes, since even random variation alone can produce substantially different intervals across studies (Amrhein et al., 2019). Thus, while not statistically significant, three other predictors bear mentioning. Firstly, running counter to our hypotheses, the odds of presenting with depression at a 12-month follow-up were decreased by 69% for participants in the control group compared to the relapse prevention treatment booster group. While not statistically significant, this difference is equivalent to a Cohen’s *d* effect size of 0.65 which is moderate. A cautious interpretation of these findings would be that the relapse prevention booster program was unsuccessful in decreasing the probability of relapse. Secondly, as predicted, males were less likely to present with depression at a 12-month follow-up; males had 96% lower odds of being depressed compared to females. While not statistically significant, this difference equates to a Cohen’s *d* effect size of 1.78 which is large. Finally, as predicted, participants who previously underwent psychotherapy were more likely to present with depression at the 12-month follow-up. Specifically, participants with a history of prior psychotherapy had 68% greater odds of relapse than those with no history of prior psychotherapy, equivalent to a Cohen’s *d* of 0.29 which is small. Other predictors had negligible effects on depression status at a 12-month follow-up.

Similar to the 12-month follow-up assessment, several predictors were notably associated with depression status at a 24-month follow-up (see Table 4). However, no predictor was as a statistically significant predictor of depression status at a 24-month follow-up.

**Table 4:** Results of the logistic regression analysis depression status at 24-month follow-up (Not-Depressed = 0, Depressed = 1).

Predictor	β	OR	*p*-value
(Intercept)	−3.95 (2.42)	0.02 [0.00; 1.98]	0.102
Treatment Group: Control	−0.26 (0.76)	0.77 [0.17; 3.49]	0.727
PHQ-9: Post-Treatment	0.12 (0.09)	1.13 [0.93; 1.38]	0.194
GAD-7: Post-Treatment	0.00 (0.12)	1.00 [0.78; 1.28]	0.980
QOLI: Post-Treatment	−0.21 (0.26)	0.81 [0.48; 1.36]	0.420
Age	0.07 (0.04)	1.07 [1.00; 1.15]	0.061
Gender: Male	−1.44 (1.06)	0.24 [0.02; 1.63]	0.177
Marital Status (descending)	−0.92 (0.68)	0.40 [0.08; 1.32]	0.176
Education Level (ascending)	−0.04 (0.52)	0.96 [0.34; 2.79]	0.933
Prior Pharmacotherapy: Yes	0.04 (0.83)	1.04 [0.20; 5.50]	0.965
Prior Psychotherapy: Yes	0.66 (0.82)	1.93 [0.40; 10.70]	0.421

Standard errors are displayed in parentheses; 95% confidence intervals are displayed in brackets. The model fit was suboptimal, χ2(10) = 15.89, p = 0.103. PHQ-9, Patient Health Questionnaire 9-item; GAD-7, Generalized Anxiety Disorder scale 7-item; QOLI, Quality of Life Inventory.

Nonetheless, while not statistically significant, three predictors bear mentioning (cf. Amrhein et al., 2019). Firstly, as predicted, males were less likely to present with depression at a 24-month follow-up; males had 76% lower odds of being depressed compared to females. While not statistically significant, this difference equates to a Cohen’s *d* effect size of 0.79 which is large. Secondly, as predicted, marital status had a notable relationship with depression status at a 24-month follow-up. Marital status was coded as a numeric variable (where being married or relationship was equal to 1, being single was equal to 2, and being divorced was equal to 3) and a change in marital status from being married or in a relationship towards being single decreased the odds of depression at a 24-month follow-up by 60%. While not statistically significant, this difference equates to a Cohen’s *d* effect size of 0.51 which is moderate. Finally, as predicted, participants who previously underwent psychotherapy were more likely to present with depression at the 12-month follow-up. Specifically, participants with a history of prior psychotherapy had 93% greater odds of relapse than those with no history of prior psychotherapy, equivalent of a Cohen’s *d* of 0.36. Other predictors had negligible effects on depression status at a 24-month follow-up.

## 4 Discussion

This study aimed to evaluate the long-term efficacy of internet-based behavioral activation and physical activity, assess factors that are predictive of relapse prevention, and evaluate the effectiveness of the relapse prevention program. In contrast to our hypothesis, we found no significant differences in relapse rates between the relapse prevention booster group and a control group as measured by the MINI diagnostic interview on a trimonthly basis for 24-months following acute treatment. Similarly, we found no significant differences between these groups on the PHQ-9 at a 12-month and 24-month follow-up, even when controlling for commonly reported confounding variables in treatment outcome studies of depression (for a review, see Webb et al., 2017). However, a significant difference in survival rates between the relapse prevention group and control group was detected that favored the control group condition when analyses were based on monthly PHQ-9 follow-up assessments. A cautious interpretation of these findings may be that the relapse prevention booster program was unsuccessful in decreasing the probability of relapse. However, the similar depression-free trends over the course of the study period, wherein over 95% of participants in both groups maintained remission at the 24-month follow-up, is notable and substantially greater than expected if depression had not been treated (Whiteford et al., 2013).

These findings suggest that the specific relapse prevention program used in this study may not have been effective in reducing the likelihood of relapse, despite previous research indicating the potential benefits of other relapse prevention strategies, such as CBT booster sessions for iCBT-naïve individuals already in a risk of relapse following MDD treatment (Holländare et al., 2011). Moreover, meta-analytic findings have suggested relapse rates to be over 20% lower among participants provided with relapse prevention programs (Bruin et al., 2022; Clarke et al., 2015). While our results align with previous findings showing no long-term benefit of booster sessions in depression treatment (Hadjistavropoulos et al., 2022; Peynenburg et al., 2022), they require cautious interpretation because attrition was substantial at the final assessment point. Specifically, previous research suggests that participants who express enthusiasm for additional therapy sessions (e.g., assignment to a booster-treatment arm) generally report lower pre-treatment symptom severity (Patterson et al., 2022). Put differently, those who most welcome a booster often have the least to gain from it, whereas individuals with greater clinical need are more likely to drop out or refuse extra sessions. As a result, the very participants who could benefit most from a booster may not have received it because of poor adherence or negative attitudes toward extended treatment.

We also hypothesized that pre-booster treatment levels of depression, anxiety, quality of life, age, education level, marital status, gender, prior psychotherapy, and prior pharmacotherapy would moderate the risk of relapse at a 12-month and 24-month follow-up assessments. Of these variables, only pre-booster treatment levels of quality of life were associated with lower risk of relapse at the 12-month follow-up assessment; quality of life was not associated with relapse at 24-month follow-up assessment. Other variables did not reach statistical significance at either the 12-month or 24-month follow-up assessment. However, the odds of relapse appeared to be greater among participants in the relapse prevention treatment booster group at the 12-month follow-up assessment, although this difference was not statistically significant and not detected at the 24-month follow-up. A cautious interpretation of these findings may be that the relapse prevention booster program was unsuccessful in decreasing the probability of relapse.

Males were found to be less likely to relapse at both the 12-month and 24-month follow-up assessments, compared to females. Although these differences were not significant, a large effect size was detected at both follow-up assessments for gender. It is important to keep in mind that statistically non-significant findings can still provide insights into clinically meaningful effects; due to random variation alone substantially different confidence intervals across studies are to be expected. Thus, whether confidence intervals include zero or not, this should not impede meaningful interpretation of effect sizes (Amrhein et al., 2019; Muff et al., 2022). A further caveat to these findings is the over-representation of females compared to males in the sample. Finally, at the 24-month follow-up assessment, marital status was moderately associated with relapse; a change in marital status from being married or in a relationship towards being single decreased the odds of depression at a 24-month follow-up by 63%. However, this association was not significant and not detected at the 12-month follow-up assessment.

Finally, a small but consistent association was detected between prior psychotherapy and relapse at the 12-month and 24-month follow-up assessments. Although this association was not statistically significant, it may hold clinical significance (Amrhein et al., 2019). Existing literature suggests that individuals that have previously undergone psychotherapy and/or pharmacotherapy for depression may suffer from “difficult-to-treat depression” (McAllister-Williams et al., 2020; Rush et al., 2022). In this study, individuals were 68% more likely to have relapsed at a 12-month follow-up and 93% more likely to have relapsed at a 24-month follow-up if they had previously undergone psychotherapy.

Taken together, our hypothesized negative association between relapse and pre-booster treatment levels of depression, anxiety, quality of life, age (higher), education level (higher), marital status (divorced), female gender, previous psychotherapy and/or pharmacotherapy were statistically unsupported by the data, although interesting associations were detected. These results contrast previous findings in the literature (Alber et al., 2023; Donker et al., 2013; Webb et al., 2017) but may concomitantly indicate the effectiveness of the treatment interventions received by participants in the treatment phase is greater than previously thought (for the treatment phase results, see Nyström et al., 2017).

### 4.1 Limitations and strengths

This study does have limitations. Notably, the MINI interview survival analyses and PHQ-9 survival analyses yielded differing results. However, these analyses had similar visual trendlines for survival rates and as the MINI diagnostic interviews are considered the “gold-standard” for diagnosing mental disorders, the totality of our study’s findings does suggest that there were no clinically significant differences between the relapse prevention treatment group and control group. Nonetheless, our findings must be interpreted in context of the differing results by measurement of depression. This study is also limited by the relatively few participants in the subgroups for marital status and education levels, making it unfeasible to compare participants who were divorced to those who were single or in a relationship. Similarly, most of the sample either had a high school or graduate school level of education; approximately 17% of participants had an elementary level of education, and 8% had a postgraduate level of education. Consequently, comparisons between subgroups for marital status and education level were not possible, as the number of participants in these subgroups did not meet the minimal requirements for subgroup sample size (cf. central limit theorem). Another limitation could be that data collection ended nearly a decade ago; however, the core components of the treatment delivery system have remained functionally unchanged and continues to be delivered in analogous text-based formats, supporting the current study’s relevance to contemporary practice. Other limitations include a disproportionately greater number of female participants compared to males, partially inconsistent results when the 12-month and 24-month follow-up assessments are compared, and increasing amount of missing data as the study progressed. Regardless, this study also has numerous strengths. Chiefly our use blinded structured diagnostic interviews, the gold standard for confirming diagnoses, buttress the validity of our findings. Additionally, the results pertaining to the diagnostic interviews were corroborated by analogous analyses with self-report data (i.e., the PHQ-9), as the diagnostic interviews and self-report data yielded isomorphic results. Other strengths include a moderately large sample size and a study design that allows for generalizability of findings between different treatment approaches (i.e., the randomization of participants to the relapse prevention treatment booster or a control group following either behavioral activation or physical activity treatment increases the generalizability of these findings).

### 4.2 Implications

Our results suggest that relapse prevention treatment booster sessions do not have a clinically significant impact on risk of relapse. However, targeting the program to specific subgroups might yield different results. For instance, preferentially recommending and providing booster sessions for first-time receivers of psychotherapy for major depression might result in different results. Future studies could investigate this by evaluating the effectiveness of booster sessions for major depression in a sample of psychotherapy-naïve sample. However, our findings may to a large extent also be a testament to the effectiveness of the treatment phase of the trial (i.e., internet-based behavioral activation and physical activity). Indeed, meta-analytic findings suggest that internet-based behavioral activation has a moderate effect size on depressive symptom severity (Alber et al., 2023; Huguet et al., 2018), consistently performs as well or better than other treatment outcomes and control groups (Fu et al., 2021; Ly et al., 2014; Nyström et al., 2015), with preliminary evidence for the maintenance of treatment gains over time (Alber et al., 2023). Similarly, the effectiveness of physical activity as a treatment option for mild–moderate depression has been documented in systematic reports and meta-analyses (Heissel et al., 2023; Hird et al., 2024; Morres et al., 2019), with reports of lasting antidepressive effects that are maintained up to 12-months following treatment (Helgadóttir et al., 2017; Wolf et al., 2024). Taken together, the fact that we did not find differences between a treatment booster group and a control group among participants that had either received internet-based behavioral activation or physical activity prior to a relapse prevention treatment group allocation may indicate that the efficacy of behavioral activation and physical activity is even greater than previously reported.

## Data Availability

The datasets for this article are not publicly available due to concerns regarding participant/patient anonymity. Requests to access the datasets should be directed to the corresponding author: per. carlbring@psychology.su.se.

## Group members of the ACTUA research group

Linn Arbeus, Annika Åberg, Jan Bergström, Samuel Bystedt, Yvette Ejdesjö, Lars Forsberg, Mikael Granlund, Peter Hassmén, Emma Hässledal, Ida Johansson, Alexander Johansson, Karin Johansson, Philip Lindner, Emilie Moberg, Moa Midvinter, Birger Moëll, Gregory Neely, Johanna Nordblom, Markus Nyström, Klas Nyrinder, Åsa Persson, Evelina Petterson, Mattias Segerlund, Anna Ruthström, Katja Sjöblom, Emma Sjöström, Natalie Svartvatten, Andreas Stenling, Lars Ström, Joel Stenfalk, Josefin Wall, Kristina Wirén, and Thomas Wigforss.
