# Supplementary material for: Evaluating the necessity of booster sessions in relapse prevention for depression: a longitudinal study
Source: Front Psychol. 2025 Jun 24;16:1568141. doi: 10.3389/fpsyg.2025.1568141 (PMC12236458; doi:10.3389/fpsyg.2025.1568141)
Supplement: Supplementary file 1 [file Table_1.docx]

Supplementary Material

Figure S1. *Flow Diagram of the Study Recruitment Process and Group Allocation.*

**
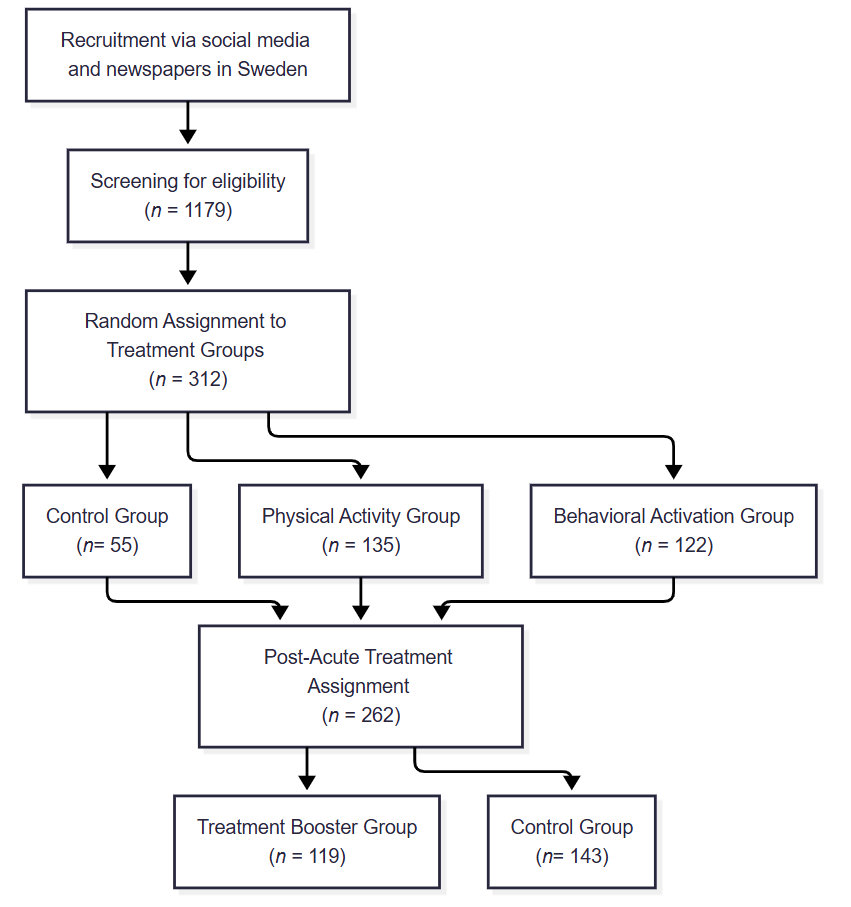
**

**Figure S1.**
*Survival Curves of Depression Status Over the Follow-Up Period by Treatment Group as Indicated by the Monthly PHQ-9 Follow-Up Assessments.*


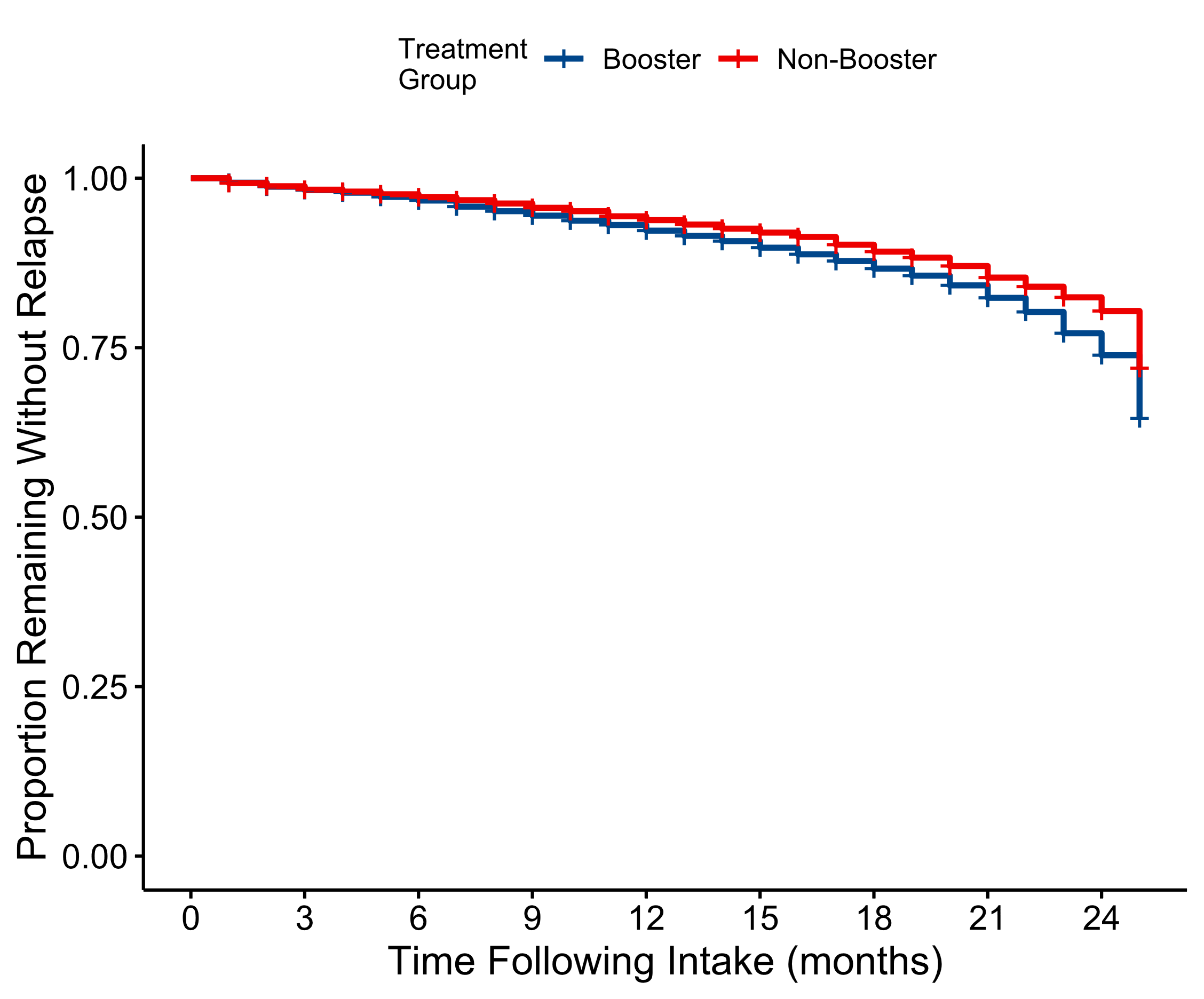


*Note.* Depression was defined as a score of 10 or higher on the PHQ-9. The difference between the relapse prevention treatment booster group (blue) and the control group (red) was statistically significant (*p* = .0013).

| **Table S1.**  *PHQ-9 Monthly Average Follow-Up Measurements.* | | |
| --- | --- | --- |
| Time Point | Booster  (*n*=119) | Control  (*n*=143) |
| Post-Assessment | 7.35 (5.19) | 7.25 (5.62) |
| Month 1 | 7.03 (5.46) | 6.50 (5.37) |
| Month 2 | 6.60 (4.56) | 5.80 (4.81) |
| Month 3 | 6.28 (4.64) | 5.57 (4.52) |
| Month 4 | 6.58 (5.08) | 5.51 (4.80) |
| Month 5 | 6.28 (4.64) | 5.93 (5.64) |
| Month 6 | 7.11 (5.85) | 6.12 (5.38) |
| Month 7 | 6.39 (5.30) | 6.01 (5.57) |
| Month 8 | 6.67 (5.32) | 6.46 (6.19) |
| Month 9 | 6.65 (5.37) | 5.85 (4.89) |
| Month 10 | 6.60 (5.01) | 6.22 (5.29) |
| Month 11 | 6.98 (5.29) | 5.88 (5.47) |
| Month 12 | 6.25 (4.97) | 5.46 (5.09) |
| Month 13 | 6.19 (5.08) | 5.51 (5.59) |
| Month 14 | 6.36 (5.38) | 5.43 (5.42) |
| Month 15 | 5.61 (4.94) | 5.29 (5.14) |
| Month 16 | 6.16 (5.74) | 6.11 (5.68) |
| Month 17 | 5.58 (5.23) | 5.38 (5.18) |
| Month 18 | 5.59 (5.50) | 5.39 (5.11) |
| Month 19 | 5.88 (5.34) | 5.23 (5.16) |
| Month 20 | 5.79 (4.99) | 5.16 (4.71) |
| Month 21 | 5.56 (4.97) | 4.54 (4.36) |
| Month 22 | 6.24 (5.78) | 4.78 (5.42) |
| Month 23 | 5.61 (5.63) | 4.38 (4.27) |
| Month 24 | 6.29 (5.24) | 5.75 (6.07) |
| *Note.* Data are presented as mean (SD). PHQ-9 = Patient Health Questionnaire 9-item. Missing data were present for both groups across all time points. For the Booster group, missing data ranged from 36 (at post-assessment) to 78 (at Month 23) participants. For the Control group, missing data ranged from 51 (at post-assessment) to 93 (at Month 23) participants. Specific numbers of missing data for each time point were as follows: Post-Assessment: 36 (Booster), 51 (Control); Month 1: 58, 67; Month 2: 51, 67; Month 3: 54, 64; Month 4: 55, 69; Month 5: 55, 71; Month 6: 55, 66; Month 7: 58, 69; Month 8: 56, 72; Month 9: 54, 68; Month 10: 56, 70; Month 11: 58, 77; Month 12: 58, 69; Month 13: 52, 72; Month 14: 60, 75; Month 15: 60, 73; Month 16: 58, 71; Month 17: 62, 71; Month 18: 63, 72; Month 19: 62, 74; Month 20: 57, 74; Month 21: 65, 73; Month 22: 56, 75; Month 23: 78, 93; Month 24: 56, 70. | | |
